# ML210 Antagonizes ABCB1- Not ABCG2-Mediated Multidrug Resistance in Colorectal Cancer

**DOI:** 10.3390/biomedicines13051245

**Published:** 2025-05-20

**Authors:** Yan-Chi Li, Yu-Meng Xiong, Ze-Ping Long, Yi-Ping Huang, Yu-Bin Shu, Ke He, Hong-Yan Sun, Zhi Shi

**Affiliations:** 1 Cancer Minimally Invasive Therapies Centre, Guangdong Second Provincial General Hospital, Jinan University, Guangzhou 510632, China; liyanchi@stu2022.jnu.edu.cn (Y.-C.L.); xymzxx@stu2022.jnu.edu.cn (Y.-M.X.); long0012@stu2022.jnu.edu.cn (Z.-P.L.); heke8@mail3.sysu.edu.cn (K.H.); 2 Department of Cell Biology & Institute of Biomedicine, Guangdong Provincial Biotechnology & Engineering Technology Research Center, Guangdong Provincial Key Laboratory of Bioengineering Medicine, Genomic Medicine Engineering Research Center of Ministry of Education, MOE Key Laboratory of Tumor Molecular Biology, National Engineering Research Center of Genetic Medicine, State Key Laboratory of Bioactive Molecules and Druggability Assessment, College of Life Science and Technology, Jinan University, Guangzhou 510632, China; 1227hyp@stu2022.jnu.edu.cn (Y.-P.H.); suyb7@jnu.edu.cn (Y.-B.S.); 3 State Key Laboratory of Oncology in South China, Guangdong Provincial Clinical Research Center for Cancer, Sun Yat-Sen University Cancer Center, Guangzhou 510060, China

**Keywords:** colorectal cancer, multidrug resistance, ABCB1, ABCG2, ML210

## Abstract

**Objectives:** ABCB1-mediated multidrug resistance (MDR) compromises chemotherapy efficacy in colorectal cancer (CRC). Despite decades of research, no selective ABCB1 inhibitor has achieved clinical success. This study investigates ML210 as a novel ABCB1-specific inhibitor to reverse ABCB1-driven MDR. **Methods:** Cytotoxicity assays (MTT) were performed on ABCB1-overexpressing HCT-8/V and ABCG2-overexpressing S1-M1-80 CRC cells. Drug accumulation (doxorubicin/mitoxantrone) was quantified via flow cytometry, and cell cycle effects were analyzed using propidium iodide staining. Molecular docking utilized the ABCB1 crystal structure. **Results:** ML210 selectively reversed ABCB1-mediated resistance to doxorubicin and vincristine in HCT-8/V cells, enhancing intracellular drug accumulation without affecting ABCG2 activity. It induced cell cycle arrest in ABCB1-overexpressing cells and did not alter ABCB1 protein expression. Molecular docking revealed stable binding of ML210 within the ABCB1 substrate pocket through hydrophobic interactions and hydrogen bonding. **Conclusions:** ML210 is a selective ABCB1 inhibitor that circumvents MDR via direct transport blockade, offering a targeted strategy against ABCB1-mediated chemoresistance in CRC. Its specificity for ABCB1 over ABCG2 highlights potential clinical advantages.

## 1. Introduction

Colorectal cancer (CRC) is the third most common cancer and represents the second primary contributor to mortality of cancer [1]. In 2022, global projections indicated approximately 1.926 million incident cases of CRC worldwide, accounting for 9.3% of all cancer diagnoses, consolidating its position as the third most common malignancy [2]. Notably, the prevalence of early-onset CRC (before the age of 50) shows an increased rate of 2%, particularly in developed nations, signaling shifting epidemiological patterns [3]. The causes of CRC include genetic factors and lifestyle. Processed meat and a low-fiber diet elevate the likelihood of developing CRC [4], while obesity, metabolic syndrome and intestinal bacterial imbalance can promote tumorigenesis [5]. Although the therapeutic outcomes of CRC have significantly improved, the 5-year survival rate of metastatic CRC (mCRC) is still less than 10%. The effective therapeutic strategies of mCRC include cytotoxic chemotherapy and targeting therapies, etc. However, around 90% of mCRCs are recurrent after treatment, mainly due to multidrug resistance (MDR) [3].

MDR is defined as the development of drug resistance that ultimately induces cross-resistance to multiple therapeutic compounds with distinct structures and pharmacological mechanisms, characterizing the reduced responsiveness of target cells to anticancer drugs [6]. As a primary cause of chemotherapy failure, MDR involves multiple mechanisms, including aberrations in drug targets, altered drug uptake and efflux, drug inactivation through enzymatic modification and enhanced DNA damage repair [7]. Among these mechanisms, overexpression of ABC transporters is closely associated with MDR in cancer. This ATP-binding cassette protein mediates ATP-dependent efflux of chemotherapeutic drugs and targeted therapeutic drugs, significantly reducing intracellular drug retention and leading to therapeutic resistance [8]. Of particular significance is the fact that overexpression of ABCB1 constitutes a crucial factor in developing MDR in cancer cells.

First-line therapeutic selection in CRC is governed by molecular profiling and clinical staging, encompassing three major modalities—chemotherapy, targeted therapy and immunotherapy—to balance efficacy and safety through precision strategies. 5-Fluorouracil (5-FU) remains the safest, most effective and widely utilized chemotherapeutic agent in CRC treatment, serving as the backbone of fluoropyrimidine-based regimens [9]. However, severe adverse effects and dose-limiting toxicities persist in a subset of patients [10]. Irinotecan has emerged as a cornerstone therapeutic agent in CRC due to its demonstrated overall survival (OS) benefits. Yet, its clinical utility is constrained by substantial interpatient and intrapatient variability in the systemic exposure of its active metabolite SN-38, which directly induces dose-limiting irinotecan-associated toxicities [11]. Oxaliplatin-based chemotherapy serves as a cornerstone regimen in advanced CRC, being primarily employed as first-line therapy and as adjuvant treatment following complete resection of primary tumors [12]. Encorafenib (a BRAF V600E mutation inhibitor) [13] and regorafenib (a multikinase inhibitor) [14] are selectively deployed to target distinct molecular aberrations or later-line therapeutic settings, whereas capecitabine (an oral fluoropyrimidine prodrug) [15] serves as an adjuvant or alternative regimen in first-line chemotherapy. The current therapeutic paradigm is transitioning from broad-spectrum cytotoxicity to molecularly targeted approaches, driven by advances in molecular subtyping. Future strategies to overcome drug resistance and improve long-term survival may involve combinatorial therapies with ABC transporter inhibitors or novel immunotherapies, aiming to synergistically enhance therapeutic precision and durability.

ABCB1 (alternatively referred to as P-glycoprotein) belongs to the B subgroup of ATP-binding cassette transporters [16,17]. ABCB1 is composed of two transmembrane domains (TMDs) and two nucleotide-binding domains (NBDs), a structural blueprint conserved in ABCG2 despite their divergent quaternary assemblies [18,19]. The TMDs are responsible for recognizing and binding substrates, while the NBDs bind and hydrolyze ATP [20]. The multidrug efflux function of ABCB1 relies on the synergistic interplay of multiple overlapping substrate-binding sites within its dynamic drug-binding pocket. The structural hallmark underpins the broad-spectrum substrate recognition capability unique to ABC efflux transporters [21]. Frequently overexpressed in various malignancies, this transmembrane protein utilizes the energy generated through ATP hydrolysis to facilitate the active extrusion of various chemotherapeutic compounds across cellular membranes, mediating MDR [22]. This presents a significant clinical challenge in cancer therapeutics, particularly in common malignancies like CRC, where ABCB1 overexpression often correlates with chemotherapy failure. Both ABCB1 mRNA and protein expression levels were markedly elevated in CRC tissues compared to adjacent non-tumorous regions [23]. ABCB1 overexpression decreases irinotecan response rate to irinotecan-based chemotherapy by 40%, shortens PFS (HR = 1.7) and elevates hepatic metastasis risk 2.1-fold, with amplified chemoresistance and metastatic effects in CD44+/CD133+ CSCs, collectively exacerbating chemoresistance and metastatic progression in CRC [24]. In CRC-resistant SW620/Ad300 cells, multidrug resistance is driven by ABCB1 overexpression. The study confirmed that CRISPR/Cas9-mediated knockout of ABCB1 completely suppresses its expression and drug efflux function, significantly increasing chemotherapeutic drug accumulation, restoring drug sensitivity and inducing apoptosis, directly confirming ABCB1 as a central therapeutic target for chemoresistance [25]. Oncogenic upregulation of MACC1 in CRC cells enhances its binding affinity to the ABCB1 promoter region and amplifies transcriptional activation, culminating in increased ABCB1 protein abundance and consequent resistance to first-line therapeutic agents [26]. ABCB1 overexpression has been widely reported to confer drug resistance in cancer. In multiple myeloma, overexpression of ABCB1/MDR1 actively pumps the proteasome inhibitor Carfilzomib out of cells via its ATP-dependent efflux pump function, markedly reducing intracellular drug concentration and thereby driving therapeutic resistance [27]. HDAC2 directly binds to the promoter region of the ABCB1 gene and reduces the level of histone acetylation in this region through deacetylation, thereby inhibiting the transcription of ABCB1. In addition, after knocking down HDAC2 by siRNA, ABCB1 expression was significantly upregulated, resulting in reduced intracellular doxorubicin accumulation and enhanced cell resistance to doxorubicin [28]. AP-2α transcriptionally represses ABCB1 by binding its promoter under physiological conditions. Histone H3 methylation/deacetylation upregulates CCAL, which epigenetically suppresses AP-2α, activating Wnt/β-catenin signaling and ABCB1 expression, culminating in CRC multidrug resistance [29]. The m6A methylation recruits IGF2BP3 to bind ABCB1 mRNA, enhancing its stability and translational efficiency to upregulate ABCB1 expression. This increases chemotherapeutic drug efflux capacity, ultimately driving MDR in cancer cells [30].

Similar to ABCB1, ABCG2 belongs to the ABC transporter superfamily [17]. Both function as pivotal efflux transporters that actively eliminate drugs, toxins and xenobiotics from brain endothelial cells through ATP-dependent mechanisms. This energy-driven transport process establishes a critical neuroprotective barrier, effectively preventing intracranial accumulation of neurotoxic substances while maintaining cerebral homeostasis by restricting the penetration of blood-borne harmful compounds. However, ABCB1 and ABCG2 exhibit marked differences in tissue distribution, substrate specificity and regulatory mechanisms [31]. During early human gestation, ABCG2 demonstrates predominant expression, potentially critical for xenobiotic defense during organogenesis. Mid-to-late gestational stages exhibit progressive ABCB1 upregulation, corresponding to enhanced fetal detoxification requirements. This temporal regulatory dichotomy implies functional compartmentalization at maternal–fetal interfaces, with ABCG2 prioritizing developmental protection and ABCB1 addressing stage-specific metabolic challenges [32]. ABCC1 and ABCG2 exhibit specialized substrate selectivity within the multidrug efflux pump superfamily. ABCC1 mediates transmembrane clearance of amphiphilic phase II conjugates, notably glutathione- and glucuronide-modified metabolites central to endogenous detoxification. In contrast, ABCG2 demonstrates preferential efflux of hydrophobic xenobiotics, including camptothecin derivatives and antifolate agents, reflecting evolutionary adaptation to extracellular cytotoxic challenges [33]. ABCB1 is transcriptionally regulated by inflammatory cytokines (e.g., TNF-α) and nuclear receptors, such as the pregnane X receptor (PXR) and constitutive androstane receptor (CAR), with its expression being pharmacologically inducible [34,35]. ABCG2, conversely, is modulated by the hypoxia-inducible factor HIF-1α and estrogen receptor (ER), exhibiting upregulated expression under hypoxic conditions or in response to hormonal stimuli [36,37]. The investigation demonstrated that ML210, a known glutathione peroxidase 4 (GPX4)-targeting compound, exhibits dual pharmacological activity by antagonizing ABCB1 function and reversing chemoresistance in CRC models with ABCB1-driven MDR. Molecular docking experiments revealed that ML210 binds to the drug-binding site of ABCB1 via its unique chemical structure, maintaining a stable conformation through hydrophobic interactions with surrounding hydrophobic amino acids. ML210 further stabilizes its binding conformation by forming π-π stacking and intermolecular halogen bonds with ABCB1. Although ML210 is a well-known selective covalent inhibitor of GPX4, this study is the first to demonstrate its specific chemical structural features enabling competitive inhibition of ABCB1.

## 2. Materials and Methods

### 2.1. Cell Culture and Reagents

ML210 (#1360705-96-9), 3-(4,5-dimethylthiazol-yl)-2,5-diphenyl-tetrazolium bromide (MTT) (#298-93-1), verapamil hydrochloride (#V4629), oxaliplatin (#M023510), doxorubicin hydrochloride (#A603456-0025) and vincristine sulfate (#2068-78-2) were obtained from Kaiwei Chemical Technology Co. (Shanghai, China), Aikon Biopharmaceutical R&D Co. (Nanjing, China), Sigma-Aldrich Trading Co. (Shanghai, China), MREDA Technology Co. (Beijing, China) and BBI Life Sciences Corporation (Shanghai, China), correspondingly. Anti-ABCB1 antibody (#sc-8313), anti-vinculin antibody (#sc-73614) and anti-β-tubulin antibody (#66031-1-lg) were ordered from Santa Cruz Biotechnology Co. (Santa Cruz, CA, USA) and Wuhan Sanying Biotechnology Co. (Wuhan, China). The ABCB1-overexpressing human colorectal cancer MDR cell line HCT8/V was generated via stepwise selection of human colorectal cancer cell line HCT-8 in increasing concentrations of vincristine. The ABCG2-overexpressing human colorectal cancer MDR cell line S1-M1-80 vector and its ABCG2-knockout cell line S1-M1-80 sgABCG2 were established by using CRISPR/Cas9 technology, as previously described [38]. All cell lines were cultured in Dulbecco’s modified Eagle’s medium containing 10% fetal bovine serum of CellMax (#SA301.02.V) from Lanzhou Minhai Bio-Engineering Co. (Lanzhou, China) under normal conditions (5% CO_2_, 37 °C, humidified atmosphere).

### 2.2. Cytotoxicity Assay

Cells in the logarithmic growth phase were seeded at 3 × 10^3^/well in 96-well plates and treated with the indicated agents for 72 h. Following treatment, 500 mg/mL MTT was added for 4 h. The supernatant was carefully removed, and 50 μL of dimethyl sulfoxide (DMSO) was added to dissolve the formazan crystals. Absorbance was measured at 570 nm (reference wavelength: 630 nm) using a BioTek Synergy H1 plate reader (Winooski, VT, USA). Three independent experiments were performed to ensure reproducibility. The Bliss method was applied to calculate IC50 values, which mathematically models drug interactions by comparing the observed and expected effects. All procedures adhered to aseptic techniques in a biosafety cabinet. Data were analyzed using GraphPad Prism 9.0, with results expressed as mean ± SD of triplicate wells.

### 2.3. Cell Cycle Assay

Cells were seeded at 1 × 10^5^/well in 6-well plates and treated with the indicated agents for 24 h. Then, cells were harvested and fixed in ice-cold 70% ethanol for 30 min. After centrifugation (200× *g*, 5 min), cells were resuspended in 200 μL staining buffer containing 50 μg/mL PI, 0.1% sodium citrate, 0.1% Triton X-100 and 100 μg/mL DNase-free RNase A at room temperature under dark conditions for 30 min. Fluorescence was measured with an FL-2 filter (585 nm emission) and a 480 nm excitation wavelength via Beckman Coulter CytoFLEX flow cytometry (Brea, CA, USA). Data analysis was performed using ModFit LT 5 software.

### 2.4. Drug Accumulation Assay

Cells were seeded at 5 × 10^4^/well in 12-well plates and pretreated with ML210 3 μM, 10 μM or verapamil 10 μM for 1 h. Subsequently, cells were exposed to doxorubicin or mitoxantrane 10 μM for 2 h to induce DNA damage. Fluorescence imaging was performed using an Olympus CKX53 microscope (Tokyo, Japan) with standardized exposure settings to capture drug uptake or apoptotic markers. Cellular samples were then processed for quantitative analysis via Beckman Coulter CytoFLEX flow cytometry (Brea, CA, USA), following standard protocols. Three independent replicates were conducted, and data were analyzed using FlowJo v10.8 software.

### 2.5. Western Blot Assay

Cells were lysed in the ice-cold buffer (0.1% sodium dodecyl sulfate, 0.5% sodium deoxycholate, 1% nonidet P 40, 10 ng/mL phenylmethanesulfonyl fluoride, 0.03% aprotinin and 1 µM sodium orthovanadate) for 30 min at 4 °C. Following centrifugation (14,000× *g*, 10 min), the clarified lysates were separated with 10% sodium dodecyl sulfate-polyacrylamide gel electrophoresis and transferred to polyvinylidene difluoride membranes. After blocking with 5% non-fat milk, membranes were sequentially probed with target-specific primary antibodies, as well as horseradish peroxidase-linked secondary antibodies. Signal bands were visualized through chemiluminescent detection using an Analytik Jena ChemiDoc XRS imaging system (Thuringia, Germany).

### 2.6. Docking Analysis

The human ABCB1 structure (PDB ID: 4Q9H) was retrieved from the Protein Data Bank. ML210-ABCB1 docking simulations were performed using AutoDock Vina 1.1, with molecular graphics generated in PyMOL 2.3.

### 2.7. Statistical Analysis

Statistical significance was determined using Student’s *t*-test (GraphPad Prism 9), with a *p*-value threshold of <0.05.

## 3. Results

### 3.1. ML210 Antagonizes ABCB1-Mediated MDR in CRC Cells

**Figure 1:**
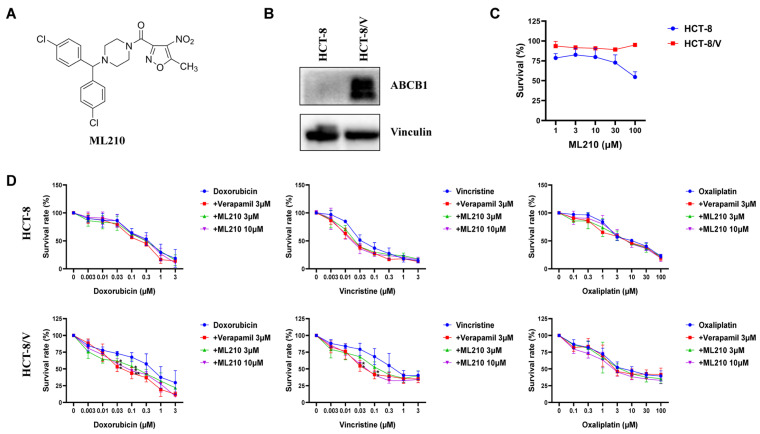
ML210 antagonizes ABCB1-mediated MDR in CRC cells. (**A**) The chemical structure of ML210. (**B**) Expression of ABCB1 proteins in HCT-8 and HCT-8/V cell lines. Cells were treated with the indicated agents for 72 h and detected by the MTT assay. Representative cell viability curves are shown (**C**,**D**). * *p* < 0.05 and ** *p* < 0.01 compared to the corresponding control groups; data are presented as mean ± SD (*n* = 3).

To elucidate the functional impact of ML210 (chemical structure presented in Figure 1A) on ABCB1-associated MDR mechanisms in CRC, we first assessed its cytotoxicity in CRC cells. ABCB1 expression levels were significantly elevated in the drug-resistant HCT-8/V cells compared with the sensitive strain HCT-8, as illustrated in Figure 1B. MTT assays showed no cytotoxicity up to 10 μM ML210 in both cells (Figure 1C), prompting the selection of 3 μM and 10 μM concentrations for chemosensitization studies. As illustrated in Figure 1D, ML210 specifically restored ABCB1-mediated drug resistance in a concentration-dependent fashion upon concurrent treatment alongside ABCB1-substrate doxorubicin as well as vincristine in HCT-8/V cells but not in HCT-8 cells, exhibiting comparable efficacy to the reference ABCB1 inhibitor verapamil. Notably, neither compound altered the sensitivity to oxaliplatin, a non-ABCB1 substrate, across both cellular models. These results indicate that ML210 antagonizes ABCB1-mediated MDR in CRC cells.

### 3.2. The Combination of ML210 with Doxorubicin or Vincristine Induces Cell Cycle Arrest in ABCB1-Overexpressing CRC Cells

**Figure 2:**
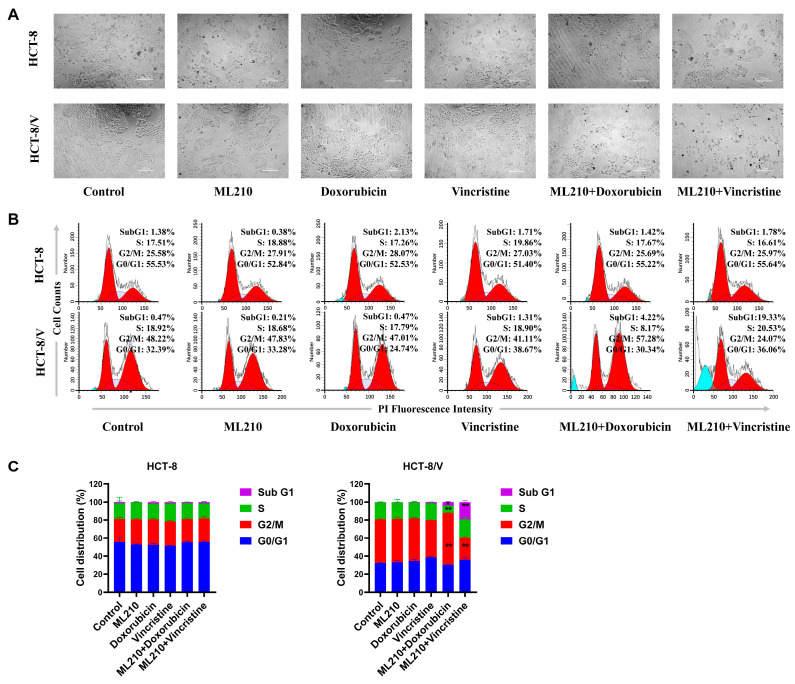
The combination of ML210 with doxorubicin or vincristine induces cell cycle arrest in ABCB1-overexpressing CRC cells. Cells were treated with the indicated reagents for 24 h, and the cell cycle distribution was analyzed by flow cytometry using propidium iodide (PI) staining. The concentrations of each agent were as follows: 10 μM ML210, 0.03 μM doxorubicin and 0.003 μM vincristine in HCT-8 cells; 10 μM ML210, 0.1μM doxorubicin and 0.1 μM vincristine in HCT-8/V cells. Representative cell morphology (**A**), histograms (**B**) and quantitative data (**C**) are shown. The scale bar is 200 μm. * *p* < 0.05 and ** *p* < 0.01 compared to the corresponding control groups; data are presented as mean ± SD (*n* = 3).

To examine the effects of ML210 combined with chemotherapeutic drugs on ABCB1-overexpressing CRC cells, we detected the cell cycle distribution following drug treatment using flow cytometry. As shown in Figure 2A–C, the combination of ML210 and doxorubicin significantly increased the sub-G1 and G2/M phase cellular fractions relative to individual treatments with either ML210 or doxorubicin in HCT-8/V cells but not in HCT-8 cells. Moreover, compared with ML210 or vincristine alone, the combined treatment of ML210 and vincristine significantly increased the sub-G1 phase and decreased the G2/M phase cell population in HCT-8/V cells but not in HCT-8 cells. These data suggest that the combination of ML210 with doxorubicin or vincristine induces cell cycle arrest in ABCB1-overexpressing CRC cells.

### 3.3. ML210 Enhances Doxorubicin Accumulation in ABCB1-Overexpressing CRC Cells and Does Not Enhance Mitoxantrane Accumulation in ABCG2-Overexpressing CRC Cells

**Figure 3:**
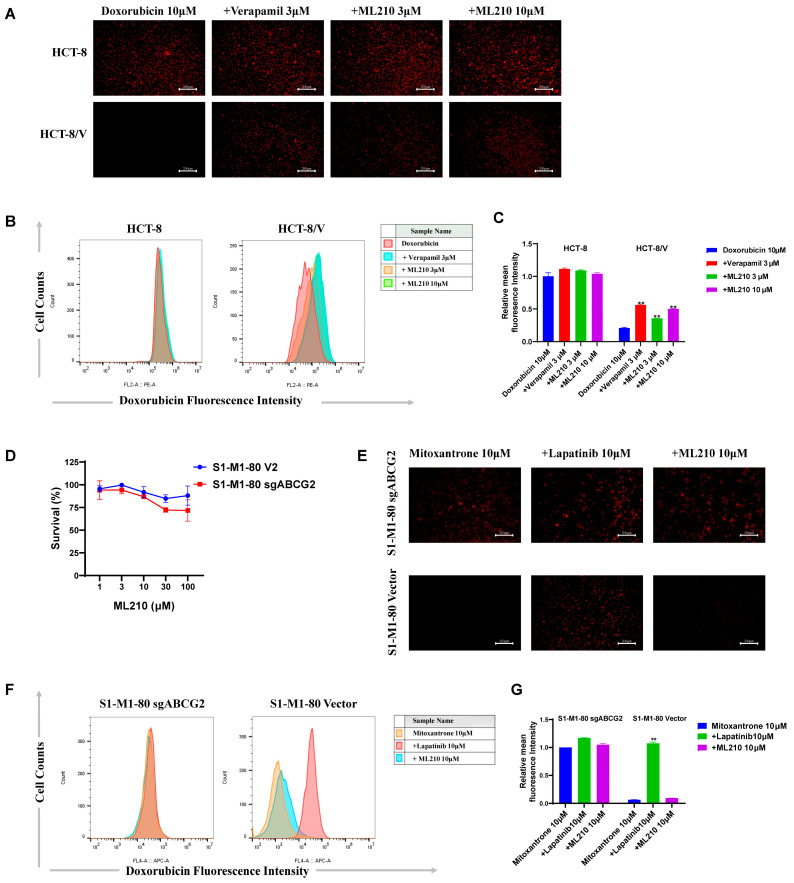
ML210 enhances doxorubicin accumulation in ABCB1-overexpressing CRC cells. After preincubation with ML210 and verapamil or lapatinib for 1 h, cells were incubated with 10 μM doxorubicin or mitoxantrone for 2 h, imaged via microscope and subsequently quantified via flow cytometry. Representative images (**A**,**E**), histograms (**B**,**F**), quantitative data (**C**,**G**) and cytotoxicity results (**D**) are shown. The scale bar is 200 μm. ** *p* < 0.01 compared to the corresponding control groups; data are presented as mean ± SD (*n* = 3).

To elucidate whether ML210 directly modulates ABCB1-mediated transport function, we performed the accumulation assays of ABCB1-substrate doxorubicin in ABCB1-overexpressing CRC cells. Resistant HCT-8/V cells, as shown in Figure 3A–C, demonstrated higher intracellular doxorubicin retention than parental HCT-8 cells. In addition, ML210 enhanced doxorubicin accumulation in HCT-8/V cells in a concentration-dependent manner, with weaker effects than verapamil at equivalent concentrations, whereas no such effect was observed in HCT-8 cells. These findings confirm that ML210 enhances doxorubicin accumulation in ABCB1-overexpressing CRC cells.

To investigate the effect of ML210 on ABCG2-mediated transport function, we examined its cytotoxicity in S1-M1-80 vector and S1-M1-80 sgABCG2 cells. As shown in Figure 3D, MTT assays showed no cytotoxicity up to 10 μM ML210 in both cells. The accumulation assays of ABC2-substrate mitoxantrane showed that the intracellular mitoxantrane in S1-M1-80 vector cells was lower than that in S1-M1-80 sgABCG2 cells. The known ABCG2 inhibitor lapatinib significantly increased the intracellular mitoxantrane in S1-M1-80 vector cells but not in S1-M1-80 sgABCG2 cells. However, 10 μM ML210 had no effect on the intracellular mitoxantrane in both S1-M1-80 vector and S1-M1-80 sgABCG2 cells (Figure 3E–G). These data demonstrated that ML210 does not enhance mitoxantrane accumulation in ABCG2-overexpressing CRC cells.

### 3.4. ML210 Does Not Affect ABCB1 Protein Levels and Its Binding Model with ABCB1

**Figure 4:**
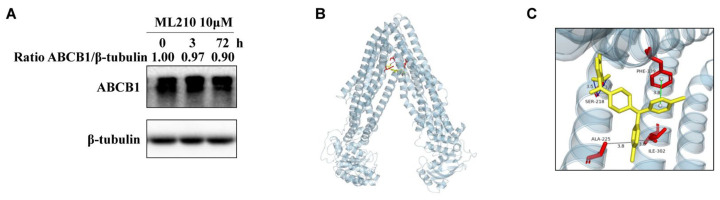
ML210 does not affect ABCB1 protein levels and its binding model with ABCB1. (**A**) ABCB1 expression levels in HCT-8/V cells treated with 10μM ML210 for the indicated time points were measured by Western blot analysis. (**B**) Optimal docking position of ML210 (red line) within the binding pocket of human ABCB1 was generated using AutoDock Vina. (**C**) A magnified view of the highlighted area shows the interaction of ML210 with ABCB1 residues Ser-218, Ala-225, Ile-302 and Phe-339.

To investigate whether ML210 modulates ABCB1 protein expression, HCT-8/V cells were treated with 10 μM ML210 for 3 and 72 h. As shown in Figure 4A, ML210 did not change the protein expression level of ABCB1. Subsequent structure-based computational analysis explored ML210–ABCB1 binding interactions. Molecular docking demonstrated ML210 occupancy within the ABCB1 substrate-binding pocket (Figure 4B,C), forming critical hydrophobic interactions with Ser-218, Ala-225, Ile-302 and Phe-339 residues to stabilize its binding conformation. Further molecular dynamic simulations identified intermolecular hydrogen bonds between ML210 and ABCB1 residue Ser-218, complemented by π-π stacking interactions with residue Phe-339, collectively enhancing complex stabilization.

## 4. Discussion

MDR is a long-standing clinical challenge in oncology therapeutics, which seriously weakens the therapeutic outcomes of chemotherapy regimens. The pathological mechanisms underlying MDR exhibit substantial heterogeneity, where hyperactivation of the ABC transporter proteins represents a prototypical contributor [39,40]. These energy-dependent efflux pumps actively expel structurally diverse chemotherapeutic agents across cellular membranes through ATP hydrolysis-derived bioenergy, resulting in intracellular drug accumulation levels substantially below therapeutic thresholds [41,42]. Notably, ABCB1, the prototypical ATP-binding cassette transporter initially identified, has been definitively associated with chemoresistance emergence across diverse cancer types [22,43]. Elevated expression levels of ABCB1 in cancer cells drive chemoresistance by facilitating the active efflux of chemotherapeutic drugs, thereby depleting their intracellular concentrations [44]. Therefore, suppression of ABCB1 expression or function may overcome the MDR phenotype. Previous studies, including our investigations, have identified multiple ABCB1 inhibitors, including small molecular compounds AG1478 [45], erlotinib [46], GSK-1070916 [47], regorafenib [48], ribociclib [49], sipholenol A [50], sildenafil [51], trametinib [52], wallichinine [53], and biotechnological strategies antibodies [54], antisense oligonucleotides [55], RNA interference [56], clustered regularly interspaced short palindromic-associated nuclease 9 [57], etc.

Our study reveals that ML210 selectively potentiates the cytotoxicity of ABCB1 substrates (doxorubicin, vincristine) in multidrug-resistant cells while exhibiting no chemosensitizing effects toward ABCG2 substrates (mitoxantrone). This target-specific modulation suggests that ML210 spares ABCG2-mediated physiological functions, thereby potentially circumventing the toxicity associated with pan-ABC inhibitors. Mechanistically, ML210 directly inhibits ABCB1 efflux activity by occupying its substrate-binding pocket without altering transporter expression levels, contrasting with indirect modulators like regorafenib and erlotinib that regulate ABCB1 transcription. These findings establish ML210 as a novel ABCB1-selective inhibitor, offering an innovative therapeutic strategy to combat MDR while minimizing off-target systemic toxicity.

ML210 is a specific covalent inhibitor targeting glutathione peroxidase 4 (GPX4) with a median effect concentration of 30 nM to induce ferroptosis [58]. In the present study, our investigation revealed ML210 as a potential therapeutic agent capable of antagonizing ABCB1- not ABCG2-mediated MDR in CRC. ML210 significantly enhances the effects of doxorubicin and vincristine on growth suppression and cell cycle perturbation in ABCB1-overexpressing CRC cells. Mechanistically, ML210 suppressed ABCB1- not ABCG2-mediated transport function and enhanced intracellular substrate retention, thereby antagonizing ABCB1- not ABCG2-mediated MDR in CRC cells. Furthermore, ML210 did not affect the expression of ABCB1 protein. Molecular docking analysis reveals that ML210 stably binds to the substrate-binding pocket of ABCB1. Interestingly, ML210 effectively inhibited migratory capacity in pancreatic ductal adenocarcinoma cells while significantly attenuating the epithelial–mesenchymal transition process when combined with the chemotherapeutic agent gemcitabine [59]. The combination of ML210 and eribulin, a microtubule inhibitor isolated from the marine sponge Halichondria okadai, had a synergistic effect on ferroptosis in ovarian clear cell carcinoma [60]. In addition, the combination of ML210 and enzalutamide, an anti-prostate-cancer drug broadly used in clinical settings that blocks the binding of androgen with its receptor, also had a synergistic antitumor effect in prostate cancer [61]. ML210 demonstrates significant antitumor efficacy in preclinical models by efficiently penetrating the blood–brain barrier (BBB), selectively inhibiting GPX4 and inducing ferroptosis in glioblastoma, resulting in a 50% reduction in tumor volume [62]. However, systemic administration may be constrained by off-target distribution and toxicity for normal tissues. Future studies should explore the delivery systems, including hydrogel, to improve the tumor-targeting efficacy of ML210 while mitigating systemic exposure risks [63]. The AGPS degradation-induced enrichment of membrane polyunsaturated fatty acids (PUFAs) synergizes with ML210-mediated GPX4 inhibition, significantly enhancing ferroptosis in prostate cancer cells [64]. In glioblastoma xenograft models, administration of ML210 as a monotherapy at 5 mg/kg significantly reduced tumor volume by 50%. When combined with temozolomide, abbreviated as TMZ, the therapeutic efficacy was further enhanced, achieving a 70% reduction in tumor volume [65]. A GPX4-targeted photosensitizer, by integrating ML210 with photodynamic therapy, reverses the hypoxic tumor microenvironment’s inhibition of ferroptosis for non-small-cell lung cancer therapy [66].

Studies have shown that cellular ferroptosis can be induced by disrupting the KIF20A/NUAK1/PP1β/GPX4 pathway, thereby overcoming oxaliplatin resistance in CRC [67]. Therefore, as a GPX4 inhibitor, ML210-induced ferroptosis may synergize with its ABCB1 inhibitory effect to combat MDR. Future studies should focus on elucidating the precise structural binding sites of ML210 on ABCB1 to optimize its selectivity. Additionally, exploring the synergistic effects of ML210 with immune checkpoint inhibitors could harness ferroptosis to enhance antitumor immunity.

## 5. Conclusions

In conclusion, ML210 can sensitize ABCB1-overexpressing multidrug-resistant colorectal cancer cells to ABCB1-substrate chemotherapeutic agents, indicating that ML210 is a promising ABCB1 inhibitor capable of antagonizing ABCB1- not ABCG2-mediated MDR in CRC.

## Data Availability

The original contributions presented in this study are included in the article. Further inquiries can be directed to the corresponding authors.
